# Considering autistic patients in the era of telemedicine: the need for an adaptable, equitable, and compassionate approach

**DOI:** 10.3399/BJGPO.2021.0174

**Published:** 2022-01-26

**Authors:** Sebastian CK Shaw, Lucy-Jane Davis, Mary Doherty

**Affiliations:** 1 Department of Medical Education, Brighton and Sussex Medical School, Brighton, UK; 2 Leatside Surgery, Totnes, Devon, UK; 3 Our Lady’s Hospital, Navan, Republic of Ireland

**Keywords:** Patient safety, inequalities, primary healthcare, general practice, telehealth, telemedicine

## Introduction

The COVID-19 pandemic has accelerated the widespread adoption of remote consultations in general practice in the UK; using telephones, video, or e-consults accompanied by a reduction in routine face-to-face consultations. These approaches allow for continued healthcare provision while reducing the risk of transmission of communicable infections. Telephone consultations have already been a staple within general practice for many years, despite a lack of high quality evidence supporting their use.^1^ Reported benefits include convenience for patients and cost savings.^2^ However, patients tend to disclose fewer medical issues on telephone calls than in face-to-face consultations.^3^ Similarly, video calls rely on high quality internet and patient ability to operate the required technology.^4^

Autism is a neurodevelopmental condition encompassing differences in social interaction, communication, and sensory perception.^5^ Differences occur in the use of verbal and non-verbal communication. Challenges can include situational mutism or reliance on augmentative and alternative communication (AAC) devices for individuals who are non-speaking. Autism is not synonymous with learning disability. In fact, the majority of autistic adults do not have a learning disability.^6^ Autism diagnoses have a prevalence of at least 1%,^7^ but autism is likely much more prevalent due to under-recognition and under-diagnosis.^7^ As such, autistic individuals are a patient group all primary care professionalsare likely to encounter on a regular or semi-regular basis. Autistic adults face increased barriers to accessing health care,^5^ and have higher mortality rates than the general population.^5^ Considering the needs of autistic patients in accessing primary health care is therefore vital if we are to embody the ethical tenets of beneficence and social justice.

We write this article as neurodivergent doctors. SS is autistic and dyslexic. He is a GP speciality trainee with a parallel academic career. He has researched and published widely around the topic of neurodiversity, with particular interests in dyslexia, dyspraxia, and autism. He is currently the research lead for Autistic Doctors International. LJD is also a GP speciality trainee, with a background in academic research and public health. She is dyspraxic and has a specialist interest in supporting neurodivergent patients. MD is an autistic consultant anaesthetist and is the founder of Autistic Doctors International. Using a combination of the wider literature and our own experiences, both as clinicians and as patients, we explore the potential strengths and limitations of telemedicine in the context of autistic patients. We also propose some suggestions for increasing its accessibility.

### Mode of consultation

Telemedicine may offer significant benefits for autistic people. Remote consultations can take place in a familiar environment, and avoid the social and sensory overstimulation experienced on public transport or in waiting rooms.^8^ Therefore the COVID-19 pandemic may have encouraged positive, inclusive change.^8^ A prior systematic review supported the use of telemedicine for autism diagnostics and interventions for autistic individuals.^9^

However, while autistic individuals have many strengths, there are also areas of life that can be more challenging. Using the telephone is difficult for many autistic adults.^5,10^ An inability to analyse non-verbal cues creates a degree of uncertainty and discomfort, which can result in reduced information transfer. Non-speaking autistic patients may need to rely on AAC or proxy communications via caregivers, which may be a particular challenge over the telephone. SS and MD personally find telephone calls anxiety-provoking. In contrast, LJD has found that telephone consultations can be used as part of a process of building a relationship with autistic patients, with a real benefit to discussing and signposting help, often alongside access face-to-face care.

Video calls may be equally challenging for autistic adults, as they *‘* *experience more stress, have less capacity to interpret verbal and non-verbal cues, and feel less empowered to participate* *’*.^11^ This has been observed in clinical practice by LJD, and it can be exacerbated by the tendency to switch within a consultation to video where it may be unanticipated as an option by the patient.

### Appointment booking

In a survey of autistic adults, 62% reported that they had difficulties booking a GP appointment by telephone, compared to only 16% of non-autistic respondents.^5^ This disparity creates issues with equitable access to primary healthcare from the outset. It is therefore important to consider how we book appointments for our patients and to consider non-telephone options such as online booking platforms.

### Appointment timing

In the triage type (or ’doctor first’) approach, where a list of calls is generated for the doctor without specific time slots, patients are less likely to be given an appointment time, but rather a general time of day for the call (‘morning’ or ‘afternoon’). This can cause specific access issues for autistic people, where they do not have time to prepare for the call or indeed suspend what they are doing for enough time to focus exclusively on that call.

### Preparation and processing

Another key aspect of remote consulting is the loss of both preparation time and the space that is normally provided by the process of attending in person. Answering a telephone call does not provide the same scaffolding. This can be exacerbated by not knowing what time to expect a call.

Communication difficulties are compounded by sensory challenges for autistic people.^5^ A supermarket or busy office can be a good example of a particularly challenging sensory environment, with bright lights, loud sounds, and many people nearby, yet patients may be unexpectedly called by their doctor while working or shopping.

The additional cognitive load and anxiety generated by a telephone call, particularly if unexpected, can leave an autistic adult unable to function as usual for some time. Moreover, during a consultation, some autistic patients may need extra time to process unanticipated questions due to difficulties in executive functioning.

### Interoception and sensory differences

Some autistic people have a reduced sense of interoception, or the perception of physiological feedback from the body.^12^ This can mean that patients have a very different experience of bodily sensations. Explaining this can be complex, and some patients may not have insight into this difference. As an anecdotal example, some patients report being very unwell with conditions that would be expected to cause significant pain, but are dismissed as they do not experience, describe, or show pain in a pattern that a doctor would traditionally recognise. This will be even more challenging during a telephone discussion for both the patient and doctor where there is an absence of physical cues, and as such we would suggest a lower threshold for face-to-face consultation.

## Summary and recommendations

We have a duty to make reasonable adjustments to facilitate access to healthcare for autistic patients. There is currently a paucity of research in this area. Table 1 outlines our recommendations for improving current practice based on our own experiences as neurodivergent patients and doctors. These provide a broad guide, but specific practice should be tailored to the needs of individual patients. By adopting simple recommendations and removing barriers, we can facilitate improved access and reduce morbidity and mortality for this vulnerable patient group.

**Table 1.** Our recommendations for autistic patients

**Reasonable adjustment**	**Rationale**
**Facilitate access**	
Patient notes should be flagged with autistic diagnosis and any specific strategies that will help for consulting	By being aware of strategies that are helpful in consulting with each patient from the outset, the clinical and supporting administrative team are better placed to offer care to their autistic patients.
Allow autistic patients to request the most accessible type of appointment for them to reduce barriers to access.	Some may not cope with telephone calls or video calls, and this may foster inequitable care.
Offer online booking	Some may not cope with telephone calls to make the booking for the appointment.
If face-to-face, offer the first or last appointment — preferably the first if a clinic is likely to run late.	Some may find the busy waiting room environment challenging and in some surgeries it will be quieter at particular times of the day.
Offer longer or double appointments.	Autistic patients may need a longer appointment because of specific social communication needs.
If remote, offer clearly defined appointment times — not a half day.	This allows autistic patients to prepare for the appointment and ensure they are in an environment which facilitates their engagement.
**During** **c** **onsultation**	
Ask the patient if they have any communication needs for you to consider at the beginning.	Autistic patients may have a variety of needs and requirements to facilitate their communication/consultation.
Consider lowering the threshold for face-to-face appointments in any triage process.	Some may find interoception challenging and thus ‘present’ in unexpected ways, which may require physical examination.
You may also wish to consider: Allowing for extra processing time between questions. Avoiding rephrasing questions unless asked to. Using clear, unambiguous language. Adding extra checks of understanding. Developing phrases which let a patient know when a question has been adequately answered and using these to help structure the consultation.	
**Following appointment**	
Use text messages to summarise information and safety-net.	This can help to ensure effective communication of key outcomes.
